# The membrane mucin MUC4 is elevated in breast tumor lymph node metastases relative to matched primary tumors and confers aggressive properties to breast cancer cells

**DOI:** 10.1186/bcr2364

**Published:** 2009-09-18

**Authors:** Heather C Workman, Jamie K Miller, Ellen Q Ingalla, Rouminder P Kaur, Diane I Yamamoto, Laurel A Beckett, Lawrence JT Young, Robert D Cardiff, Alexander D Borowsky, Kermit L Carraway, Colleen Sweeney, Kermit L Carraway

**Affiliations:** 1 Division of Basic Sciences, UC Davis Cancer Center, 4645 2ndAvenue, Sacramento, California 95817, USA; 2 Department of Dermatology, UC Davis School of Medicine, 4645 2nd Avenue, Sacramento, California 95817, USA; 3 Division of Biostatistics, UC Davis School of Medicine, Medical Sciences Building 1-C, Davis, California 95616, USA; 4 Center for Comparative Medicine, Department of Pathology and Laboratory Medicine, UC Davis, County Road 98 and Hutchison Drive, Davis, California 95616, USA; 5 Department of Cell Biology and Anatomy, University of Miami School of Medicine, 1550 NW 10th Avenue, Miami, Florida 33101, USA

## Abstract

**Introduction:**

Previous studies indicate that overexpression of the membrane-associated mucin MUC4 is potently anti-adhesive to cultured tumor cells, and suppresses cellular apoptotic response to a variety of insults. Such observations raise the possibility that MUC4 expression could contribute to tumor progression or metastasis, but the potential involvement of MUC4 in breast cancer has not been rigorously assessed. The present study aimed to investigate the expression of the membrane mucin MUC4 in normal breast tissue, primary breast tumors and lymph node metastases, and to evaluate the role of MUC4 in promoting the malignant properties of breast tumor cells.

**Methods:**

MUC4 expression levels in patient-matched normal and tumor breast tissue was initially examined by immunoblotting lysates of fresh frozen tissue samples with a highly specific preparation of anti-MUC4 monoclonal antibody 1G8. Immunohistochemical analysis was then carried out using tissue microarrays encompassing patient-matched normal breast tissue and primary tumors, and patient-matched lymph node metastases and primary tumors. Finally, shRNA-mediated knockdown was employed to assess the contribution of MUC4 to the cellular growth and malignancy properties of JIMT-1 breast cancer cells.

**Results:**

Immunoblotting and immunohistochemistry revealed that MUC4 levels are suppressed in the majority (58%, p < 0.001) of primary tumors relative to patient-matched normal tissue. On the other hand, lymph node metastatic lesions from 37% (p < 0.05) of patients expressed higher MUC4 protein levels than patient-matched primary tumors. MUC4-positive tumor emboli were often found in lymphovascular spaces of lymph node metastatic lesions. shRNA-mediated MUC4 knockdown compromised the migration, proliferation and anoikis resistance of JIMT-1 cells, strongly suggesting that MUC4 expression actively contributes to cellular properties associated with breast tumor metastasis.

**Conclusions:**

Our observations suggest that after an initial loss of MUC4 levels during the transition of normal breast tissue to primary tumor, the re-establishment of elevated MUC4 levels confers an advantage to metastasizing breast tumor cells by promoting the acquisition of cellular properties associated with malignancy.

## Introduction

Mucins comprise a large family of cell surface and secreted proteins most commonly expressed by epithelial cells [1], but they are also associated with other cell types such as the endothelial lining of vascular spaces [2,3]. Mucins are present on the apical surface of epithelial cells of gastro-intestinal, respiratory, breast, and reproductive tissues, and contribute to tissue lubrication, hydration, and protection. Mucins are defined by a serine/threonine-rich region within their extracellular domains that is heavily O-glycosylated, and the abundant O-linked glycans are largely responsible for the physico-chemical properties of mucins that contribute to epithelial protection [4,5]. It has recently become appreciated that a subset of these proteins, the membrane mucins that are physically tethered to the plasma membrane via a transmembrane domain, are capable of stimulating intracellular signaling pathways to contribute to cellular growth regulation [6-8].

MUC4, a membrane mucin, is a non-covalently linked heterodimeric protein complex composed of the two subunits MUC4α and MUC4β arising from a single transcript. The enormous extracellular MUC4α subunit contains an O-glycosylation domain and a nidogen-related domain, followed by an AMOP domain towards the C-terminus. Glycans attached to repeating units within the O-glycosylation domain of the MUC4α subunit dominate the mass of MUC4, and contribute to its protective and anti-adhesive properties. The much more modest-sized MUC4β transmembrane subunit contains a von Willebrand factor D domain, and three epidermal growth factor-like domains that lie N-terminal to the transmembrane domain; these domains may be involved in protein-protein interactions that contribute to MUC4 function [9-11]. A function for the short (about 20 amino acids) cytoplasmic tail of the MUC4β subunit has yet to be described [12].

MUC4 expression has been reported in a variety of well-differentiated epithelial tissues in the adult including gastrointestinal tract, breast [13,14], and lung [15,16]. MUC4 expression has also been reported in a variety of carcinomas including ovarian [17,18], lung [15,19], pancreatic [20,21], gall bladder [22], and breast [23]. These observations are significant because MUC4 has been demonstrated to potentiate signaling by ErbB2 [9,11], a receptor known to contribute to the malignancy of breast and ovarian tumors, as well as other tumor types. In addition, the anti-adhesive [24] and anti-apoptotic [12,25] properties of overexpressed MUC4 could provide tumor cells with a selective growth or survival advantage. Indeed, ectopic overexpression of rat MUC4 in a human melanoma model cell line increased primary tumor growth [25] and metastasis [26] efficiencies when introduced into nude mice.

Although work examining the impact of MUC4 on model tumor cell properties strongly supports the notion that the mucin can promote tumor progression, evidence that it might do so in human tumors has been harder to obtain. For example, while many studies document MUC4 expression in tumors, often analysis of matched normal tissue is lacking, raising questions as to the extent to which MUC4 is dysregulated in tumors. Moreover, the interpretation of expression studies has been hampered by the use of incompletely characterized antibodies that may not be entirely specific for MUC4. Here we develop a reliable reagent for the assessment of MUC4 expression in human tissues, and apply it to examine MUC4 expression in normal breast tissue, as well as in primary tumors and lymph node metastases. Unexpectedly, we find that MUC4 expression tends to be reduced in primary tumors relative to normal tissue, but is regained upon metastasis. Thus, re-expression of MUC4 by metastasizing cells could significantly augment their malignancy. Indeed, we further demonstrate that the presence of endogenous MUC4 in a cultured breast tumor line derived from a pleural metastasis promotes cell migration, proliferation and resistance to anoikis.

## Materials and methods

### Cell lines and cell culture

Human breast cell lines MCF10A, MCF7, MDA-MB-453, MDA-MB-435, MDA-MB-468, MDA-MB-231, MDA-MB-361, SKBR3, T47D, BT474, rat mammary tumor cell line MATB-III, and HEK293T cells were purchased from the American Type Culture Collection and cultured in their recommended media (Mediatech, Manassas, VA, USA). The JIMT-1 human breast cancer cell line [27] and its MUC4 knockdown derivative [12] have been previously described. A375-Rep8 and MCF7-Rep5 cells inducibly expressing rat MUC4 have been previously described [11,24]. Construction of the human breast cell line MCF10A-h MUC4/Y inducibly expressing the human MUC4/Y variant will be described elsewhere (Workman et al., in preparation).

### Immunoblotting experiments

Primary antibodies were from the following sources: anti-MUC4 mouse monoclonal antibody 1G8s was used as conditioned media from hybridoma line #2D10, clone HL1718, and 1G8c was purchased from Zymed (Carlsbad, CA, USA); anti-MUC4 antibodies 8G7, P-20 and H-300 were from Santa Cruz Biotechnology, Inc. (Santa Cruz, CA, USA); anti-actin and anti-α-tubulin were from Sigma (St. Louis, MO, USA). Horseradish peroxidase-conjugated secondary antibodies were from Invitrogen (Carlsbad, CA, USA), and SuperSignal West developing chemicals were from Pierce (Rockford, IL, USA). An Alpha Innotech (San Leandro, CA, USA) imaging station with FluorChem software was used to capture and quantify images.

### Human breast tissue analysis

Fresh frozen human tissues from clinical samples were provided by the National Cancer Institute Cooperative Human Tissue Network and the National Cancer Institute-funded UC Davis Cancer Center Biorepository, and were used in western blotting experiments. All of the samples were approved for laboratory use by the Institutional Review Board of the UC Davis School of Medicine. Samples were homogenized in 10 μl T-Per (Pierce, Rockford, IL, USA) per mg of tissue in the presence of 4 μg/ml leupeptin, 4 μg/ml pepstatin, 4 μg/ml aprotinin, and 100 nmol 4-(2-aminoethyl) benzenesulfonyl fluoride, and then centrifuged to remove insoluble products. Cleared lysates were added to sample buffer and analyzed by immunoblotting.

### Specificity of immunohistochemical immunoreactivity

Immunoreactivity was compared in cell lines expressing and not expressing MUC4. MCF10A-hMUC4/Y and A375-rRep8 cells stably expressing inducible MUC4 were treated with 100 ng/ml and 2 μg/ml tetracycline (Sigma, St. Louis, MO, USA) to induce and repress expression, respectively. JIMT-1-pSuper-shRNAi-hMUC4 and JIMT-1-pSuper-shRNAi-scramble stably transduced cell lines [12] were compared with assess endogenous MUC4 expression. In each case, cells grown to about 70% confluency were scraped, pelleted by centifugation, fixed in 10% buffered formalin for one hour, stored in 70% ethanol and ultimately paraffin embedded.

### Tissue micro-array staining

Unstained human tissue micro-array (TMA) slides BR451, BR480, BR481, BR701, BR721, BR722, BR801, BR1001, and BR1003 (with no overlapping cases) were purchased from US Biomax (Rockville, MD, USA). TMA samples had a core size of 1 to 2 mm and each core had a thickness of 5 μm. Matched assays containing normal tissues typically consisted of adjacent uninvolved tissue taken approximately 1.5 cm from primary tumor. Unmatched normal breast tissue came from patients of good health. TMAs were prepared as suggested by the manufacturer using the UltraVision LP Detection System (Thermo Scientific, Pittsburgh, PA, USA). Slides were deparaffinized with xylene and rehydrated with alcohol. Slides were incubated in 3% H_2_O_2 _(in deionized water) for 10 minutes to suppress endogenous peroxidase activity. Antigen retrieval was carried out by incubating slides in 1 mM ethylenediaminetetraacetic acid (EDTA), pH 8.0, for 15 minutes at 98°C. Slides were incubated for 1.5 hours with 1:25 1G8s, and counterstaining was performed using ImmunoMaster Hematoxylin (American MasterTech, Lodi, CA, USA). Immunoreactivity levels were assigned a value on a 0 to 3 scale (see below). Images were captured on an Olympus BX-40 using DP2-BSW software (Center Valley, PA, USA).

### Expression analysis

Samples were examined by the primary author (HCW) and two additional pathologists/authors (ADB and RDC). Results were compiled and statistics were provided by an author statistician (LAB). Paired samples (normal vs. primary tumor or primary tumor vs. metastasis) were analyzed in two complementary ways. First, the fraction of times one sample within a pair stained more intensely exceeded 50% was tested; McNemar's test was used, excluding cases where both stained equally, and calculated the exact two-sided binomial probability for a disparity as extreme or more extreme if the true proportion were 0.5. Next, the difference in the mean staining score was tested, and the staining levels 0, 1+, 2+ and 3+ were treated as scale values and a paired t-test to test whether the mean difference was zero was used. All tests were two-sided at level 0.025, to ensure experiment-wise error rate below 0.05 that allowed for two comparisons (normal to primary tumor; primary tumor to metastasis). Individual data samples for immunoreactivity intensity from patients with tumor, normal tissue, or metastatic tumor were also compared. For these groups of patients, we carried out independent samples t-tests to compare the mean immunoreactivity levels between groups (patients with normal vs. those with primary tumor; patients with primary tumor vs. those with metastatic tumor). Again, all tests were two-sided at level 0.025.

### Migration assay

JIMT-1 scramble and knockdown cells were seeded in triplicate at 4 × 10^4 ^cells per well in 24-well Boyden chambers with 8 μm pore polycarbonate membranes (Corning, Corning, NY, USA), using complete media in the upper chamber and serum starve media in the lower chamber. After 16 hours, the lower chamber was replaced with complete media and the upper chamber replaced with serum starve media. Cells were allowed to migrate for 18 hours, filters were fixed, stained using the Diff-quik system (Dade Behring, Newark, USA), and photographed using an Olympus DP70 200× objective and DP Controller software (Center Valley, PA, USA). Three fields of view for each well were quantified by counting all the cells in each field and averaging the results for each condition.

### Anoikis and cell cycle analyses

JIMT-1 derivatives were plated in ultra-low attachment 60 mm or 100 mm flat-bottom plates (Corning, Corning, NY, USA) in complete media containing 1% methyl cellulose (Sigma, St. Louis, MO, USA), and grown for 96 hours. Suspended cells were then collected by centrifugation, washed in PBS, and fixed in 4% paraformaldehyde for one hour followed by 70% ethanol for one hour to overnight at 4°C. Cells were then rinsed in PBS and incubated for 30 minutes in propidium iodide solution (Sigma, St. Louis, MO, USA), EDTA, RNAse, and spermine (Sigma, St. Louis, MO, USA) SubG1 and cell cycle analysis of 15,000 to 30,000 cells per sample was carried out using a Becton-Dickinson fluorescent-activated cell sorting scanner using Cellquest software by Becton Dickinson (Oakville, Ontario, Canada) and ModFit software vy Veity (Topsham, ME, USA). Anoikis data is presented as a Forest meta-analysis plot [12]. Odds ratios were calculated using the sub-G1 positive and non-positive populations for cells harboring scrambled and MUC4 knockdown shRNAs and plotted using Graphpad Prism software (La Jolla, CA, USA).

## Results

### Identification of the 100 kDa MUC4β subunit using monoclonal antibody 1G8

**Figure 1:**
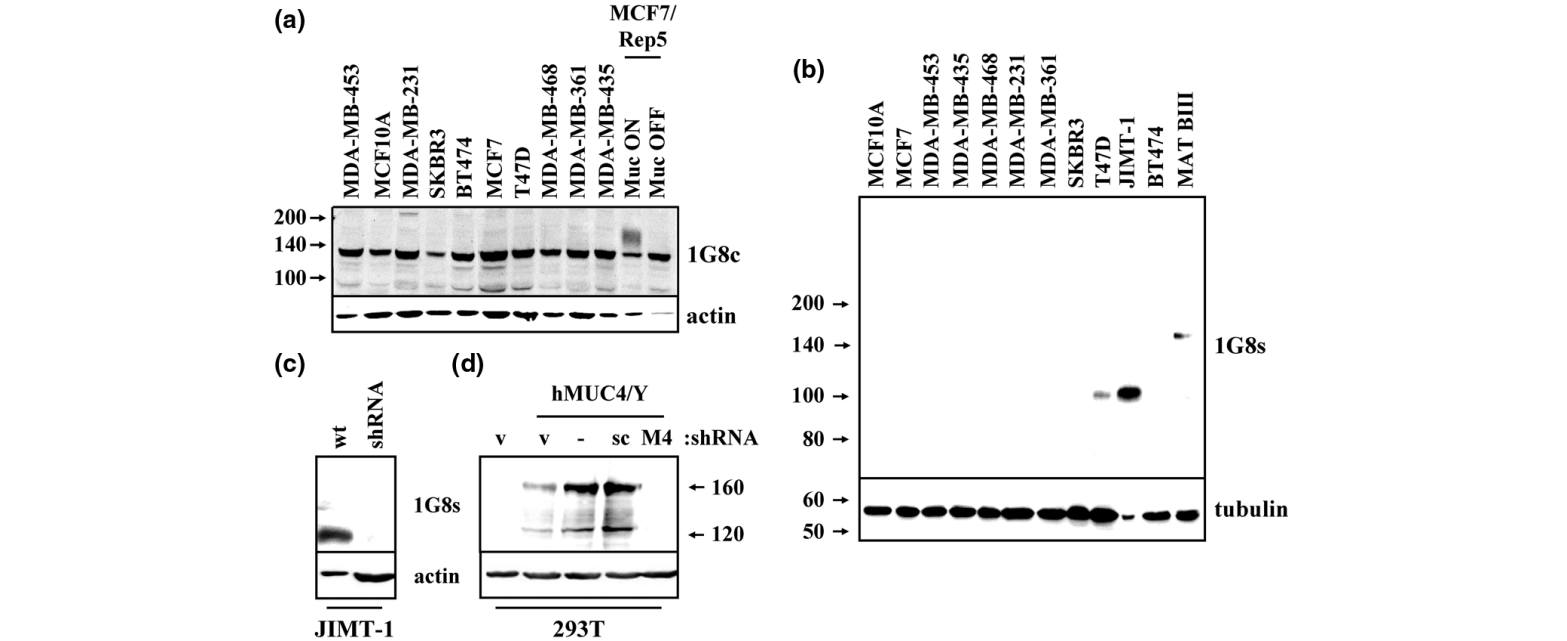
Characterization of 1G8 antibodies. **(a) **Whole cell lysates from the indicated breast epithelial cell lines were resolved by 8% SDS-PAGE and blotted with commercially obtained anti-MUC4 antibody 1G8 (1G8c). MCF7-Rep5 is a human breast cancer line stably expressing inducible rat MUC4 (24). **(b) **Cell lysates were resolved by 6% to 12% gradient SDS-PAGE and immunoblotted with supernatant from cultured 1G8 hybridoma (1G8s) or with anti-tubulin. The MATB-III rat mammary adenocarcinoma cell line was used as a positive control for MUC4 expression (12, 44, 45). **(c) **Wild-type JIMT-1 or JIMT-1 cells stably transduced with MUC4 shRNA were blotted with 1G8s and actin. **(d) **HEK293T cells were transiently co-transfected with pLenti vector control or pLenti-hMUC4/Y vector, together with pSuper vector control (v) or MUC4 shRNA (M4) or scrambled (scr) oligonucleotides in pSuper. Lysates were blotted with 1G8s and actin.

In an effort to identify an antibody that would be useful in assessing MUC4 expression in human breast tissue samples, we first screened a spectrum of antibodies for their abilities to specifically immunoblot MUC4 in cultured breast cell lines. We observed that many commercially available antibodies, including H-300, P20, and 8G7, exhibited marginal specific blotting and/or multiple background bands (not shown). Monoclonal antibody 1G8, raised to the beta subunit of rat MUC4 [28], has been previously reported to detect human MUC4 in various tissues and cell lines [2,3,12,29]. However, we observed that 1G8 obtained from a commercial source, which we call 1G8c, recognized a very prominent non-specific band of about 135 kDa in all cell lines, but only weakly recognized rat MUC4 inducibly expressed in human MCF7 breast cancer cells (Figure 1a). The reactivity of these antibody preparations with unrelated proteins calls into question results that might be obtained with their use in the immunohistochemical characterization of tissue samples.

In contrast, conditioned media from the 1G8 hybridoma, which we call 1G8s, recognized a single band of about 100 kDa that was prominently expressed in JIMT-1 cells, modestly expressed in T47D cells (Figure 1b), and expressed at very low levels in SKBR3 and BT474 cells (not shown). No evidence of the non-specific band of about 135 kDa was observed with 1G8s. As expected, 1G8s recognized a single band of about 145 kDa in rat 13762 MATB-III mammary tumor cells, corresponding to the heavier rat MUC4β form to which the antibody was originally raised [28]. Importantly, the band of about 100 kDa was lost when human JIMT-1 cells were stably transduced with MUC4-directed shRNA in a retroviral vector (Figure 1c). Finally, 1G8s recognized a pair of bands at about 160 kDa and about 120 kDa in 293T cells transiently transfected with human MUC4/Y, an alternatively spliced form of MUC4 lacking much of the alpha subunit [20,30,31]. These bands were also suppressed by MUC4-directed shRNA (Figure 1d), and most likely correspond to uncleaved MUC4/Y and MUC4/Y cleaved into its beta and residual alpha subunits. Together, these results suggest that the 1G8s preparation specifically recognizes human MUC4 in breast cancer cells, and will be useful in the analysis of human breast tissue samples.

### Loss of MUC4 expression in primary breast tumors

**Figure 2:**
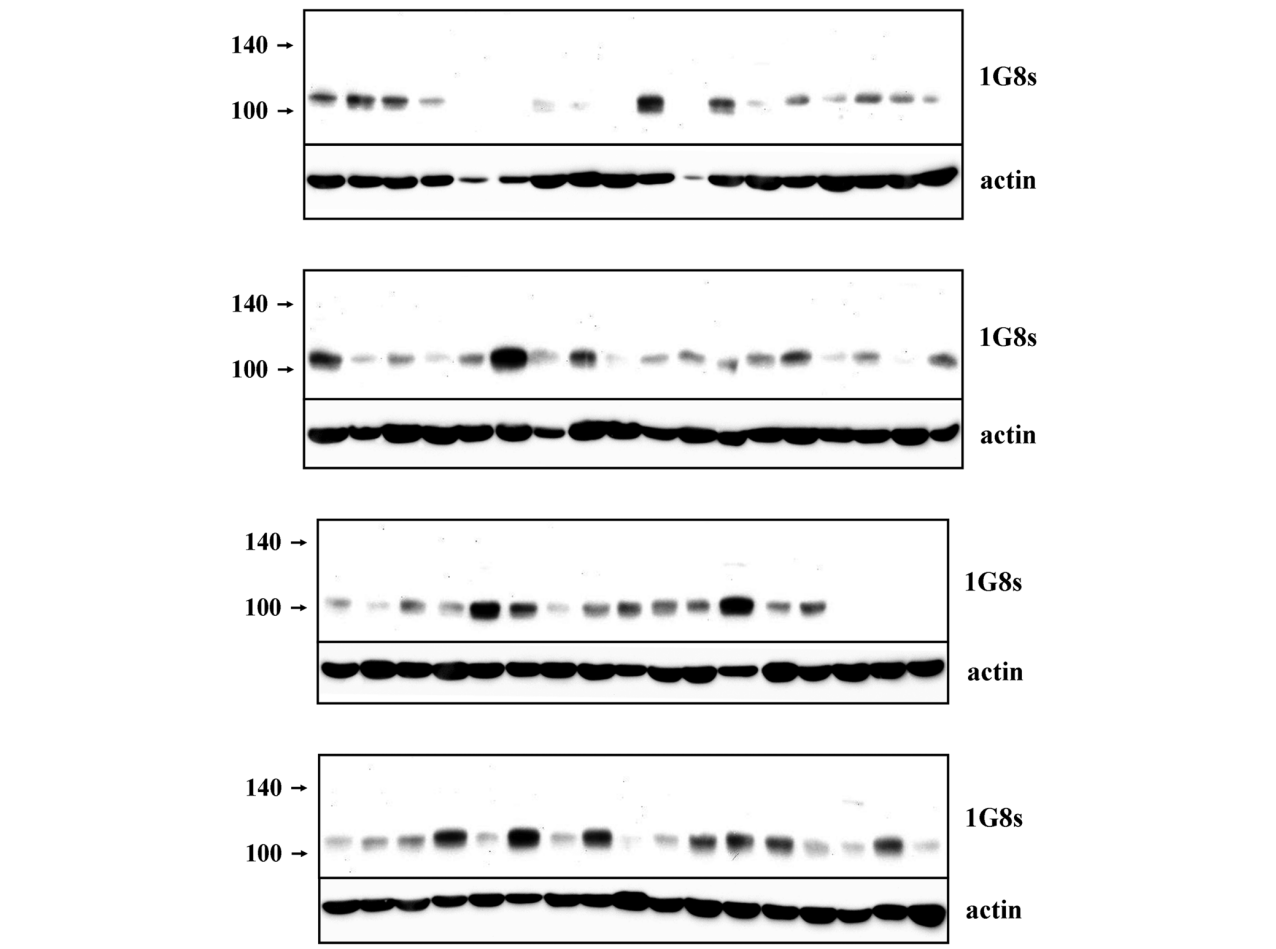
Presence of MUC4β in primary human breast tumors. Tissue lysates from 70 human primary breast tumors were immunoblotted with 1G8s and actin.

To determine whether MUC4 expression is dysregulated in human breast tumors, we first immunoblotted tissue lysates of primary tumor samples from breast cancer patients with the 1G8s preparation. Consistent with a previous report that concluded that MUC4 is expressed in 95% of breast cancers on the basis of immunohistochemical staining [23], we detected the presence of the MUC4β band of about 100 kDa in 59 of 70 (84%) of tumors (Figure 2), although the relative levels of MUC4β protein varied considerably among tumor samples. Moreover, because only modest amounts of material were available for some samples, reflected in low actin content by immunoblotting, the figure of 84% must be considered to be a lower limit for the extent of Muc4 expression in breast tumors.

**Figure 3:**
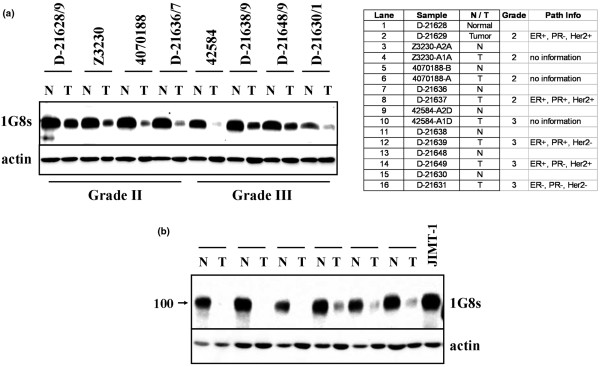
MUC4 expression is consistently suppressed in the normal-to-tumor transition. **(a) **Lysates from eight patient-matched normal and tumor tissue samples, four representing grade II tumors and four representing grade III tumors, were immunoblotted with 1G8s and anti-actin (right panels). Pathology analysis of patient tumor samples, including estrogen receptor (ER), progesterone receptor (PR) and human eipdermal growth factor receptor (HER) 2 status, are provided in the table. **(b) **Lysates from six independent patient-matched normal and tumor tissues were analyzed side by side with JIMT-1 cell lysates.

Unexpectedly, however, when we compared patient-matched normal and tumor tissue by immunoblotting, we observed a marked decrease of MUC4β protein in all tumors relative to adjacent normal tissue. In the experiments illustrated in Figure 3, a total of 14 patient-matched tumor and adjacent normal tissues were compared by immunoblotting with 1G8s. Loss of MUC4β was a common feature of all tumors surveyed, and appeared to be independent of tumor grade, or estrogen receptor (ER), progesterone receptor (PR) or human epidermal growth factor receptor (HER) 2 status (Figure 3a). Some sample pairs were run side by side with lysates from JIMT-1 cells (Figure 3b), demonstrating that the band blotted in tumors is identical in migration to the band of about 100 kDa that can be knocked down with MUC4-directed shRNA. These observations indicate that the loss of MUC4β protein may be a feature common to the transition from normal mammary epithelial tissue to tumor, and are consistent with a model whereby MUC4 protein expression is suppressed upon dedifferentiation of epithelial cells.

### Immunohistochemical analysis of human breast tumors

**Figure 4:**
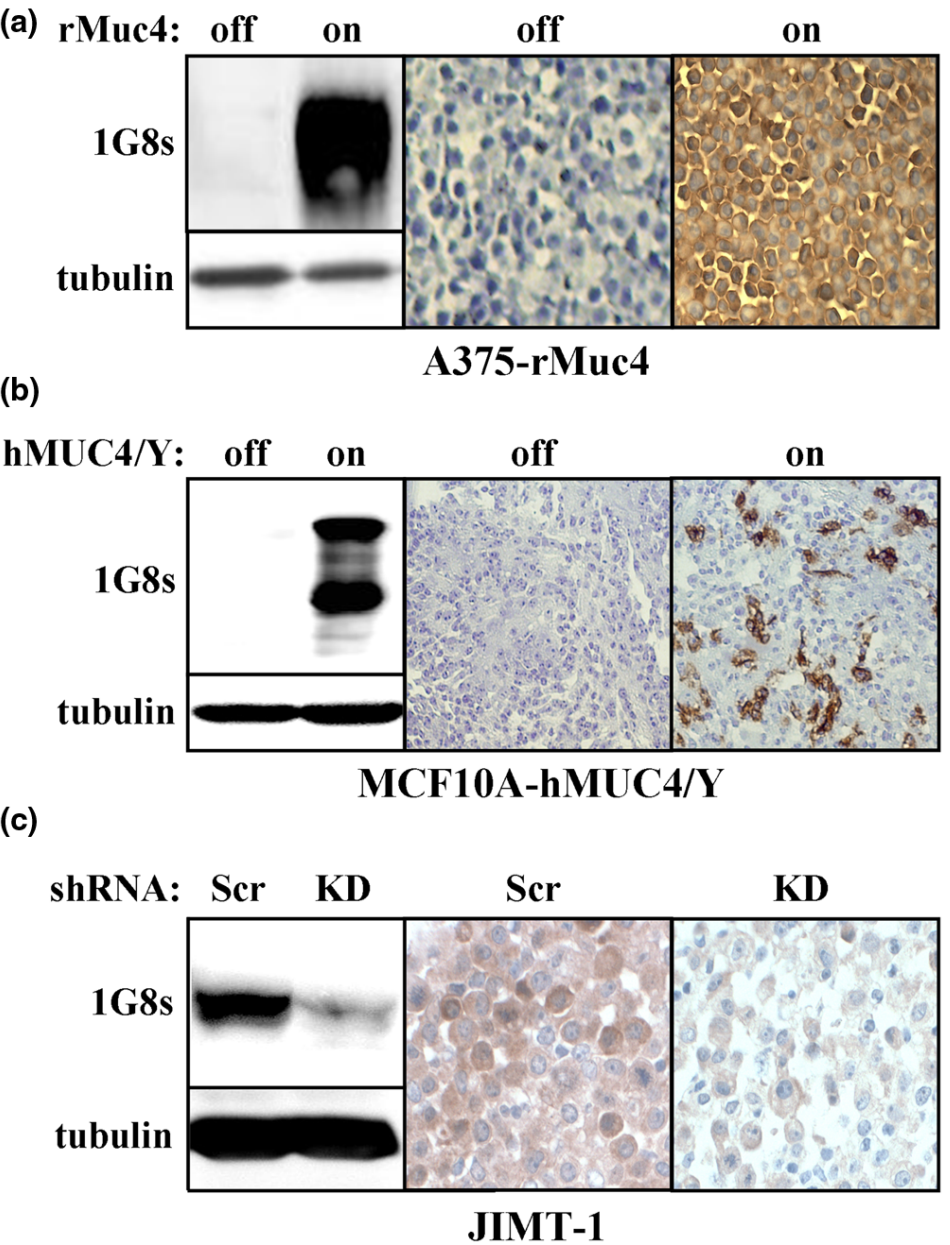
Characterization of 1G8s for immunohistochemistry. **(a) **A375-Rep8 cells (24) were treated with or without tetracycline to induce Muc4 expression, and cells were harvested and lysates were blotted with 1G8s and anti-tubulin (left panels), or cells were fixed in formalin, embedded in paraffin and analyzed by immunohistochemistry using 1G8s. **(b) **MCF10A-hMUC4/Y cells were treated with or without tetracycline to induce Muc4 expression, lysed and blotted with 1G8s and anti-tubulin (left panels), or analyzed by immunohistochemistry using 1G8s. **(c) **JIMT-1 cells stably transduced with MUC4-directed shRNA (KD) or scramble (scr) were blotted with 1G8s and anti-tubulin (upper panels), or analyzed by immunohistochemistry using 1G8s.

To examine patterns of MUC4 protein expression in breast tumors, we first optimized the 1G8s antibody preparation for immunohistochemical reactivity toward MUC4-expressing cultured cells. Under optimal conditions 1G8s stained rat MUC4 inducibly expressed in A375 melanoma cells (Figure 4a), revealing a uniform cell surface expression pattern in all cells of the field. In addition, 1G8s stained inducible human MUC4/Y stably expressed in MCF10A cells (Figure 4b). Finally, 1G8s staining of endogenous MUC4 in JIMT-1 cells was markedly diminished when MUC4 expression was knocked down (Figure 4c). These observations strongly suggest that 1G8 specifically recognizes MUC4 by immunohistochemistry.

**Figure 5:**
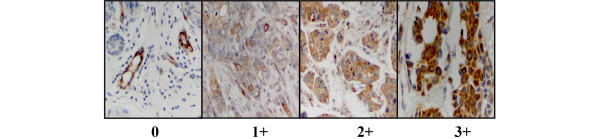
1G8s staining of breast tumor samples, illustrating examples of the 0 to 3+ staining scale employed.

To assess MUC4 expression in breast tissue, we examined nine commercially obtained TMAs encompassing over 600 samples of individual and patient-matched normal tissue, primary tumor and lymph node metastases. Immunohistochemical staining of each sample by 1G8s was assigned a score of 0 to 3+ as follows: 0, no stain to less than 30% of cells staining faintly; 1+, greater than 30% of cells staining light to moderate; 2+, greater than 50% of cells staining moderately; 3+, intense staining of majority of the epithelial population (Figure 5). Blood vessels served as internal positive controls, because it has been previously shown that endothelial cells express abundant MUC4 [2,3]. Samples whose vessels stained negative or only faintly positive were not included in the statistical analysis.

Table 1 summarizes our analysis, listing sample types and average MUC4 staining intensities; the upper tier represents our analysis of individual samples, while the lower tier represents our analysis of patient-matched paired samples. In general, there was very good agreement in average MUC4 staining intensity of normal, primary tumor, and metastatic tissue between the two groups. Consistent with our immunoblotting results, MUC4 expression was significantly greater (*P *< 0.001) in normal tissue than primary tumor, whether looking at individual or paired samples. In the paired samples, 57.5% of primary tumors exhibited suppressed MUC4 levels relative to patient-matched normal tissue, while 11% overexpressed mucin. The intensity and pattern of MUC4 staining in primary tumors was not significantly associated with HER2, ER, PR, or p53 status, nor was it associated with tumor stage or grade (not shown). Importantly, in both individual and paired analyses MUC4 expression was significantly higher (*P *< 0.05) in metastatic lesions than in primary tumors, suggesting that MUC4 re-expression may be common to breast tumor metastasis. In the paired samples, 37.2% of lymph node metastases expressed higher MUC4 levels than patient-matched primary tumors, while only 9.3% expressed lower levels. These observations point to a strong tendency for metastasized breast tumors to overexpress MUC4 relative to primary tumors, perhaps pointing to a functional role for MUC4 in tumor progression.

**Table 1:** Muc4 expression in human breast tissue by immunohistochemistry with 1G8s. Tissue samples from normal breast tissue, primary tumors or lymph node metastases were assigned a value of 0 to 3+ based on their MUC4 staining intensity with 1G8s. Statistical analysis of MUC4 staining intensities is presented for individual samples (upper panel) and patient-matched samples (lower panel).

Tissue type - individual	Number of cases (%)^a^	Composite score (mean ± standard error)	*P *value Norm vs Prim	*P *value Prim vs Met
Normal	110 (92.4)	2.01 ± 0.09	< 0.001	
Hyperplastic	26 (100)			
Ductal carcinoma *in situ*	14 (85.7)			
Primary tumor^*b*^	264 (79.5)	1.30 ± 0.06		
Primary lobular carcinoma	22 (68.1)			
Metastasis	48 (84)	1.63 ± 0.15		0.033
**Tissue type - paired**	**Number of pairs**	**Composite score (mean ± standard error)**	***P *values McNemar's test**	
Normal with matched primary	73		< 0.001	
Normal		2.07 ± 0.10		
Primary		1.28 ± 0.12		
Primary with matched metastasis^*c*^	43		0.0025	
Primary		1.10 ± 0.21		
Metastasis		1.95 ± 0.41		

*a*. Percent of patient samples that had 1+ stain or greater on a scale from 0 to 3+.

*b*. Primarily consists of invasive ductal carcinoma-NOS, but also includes invasive lobular carcinoma, micropapillary and mucinous carcinomas.

**Figure 6:**
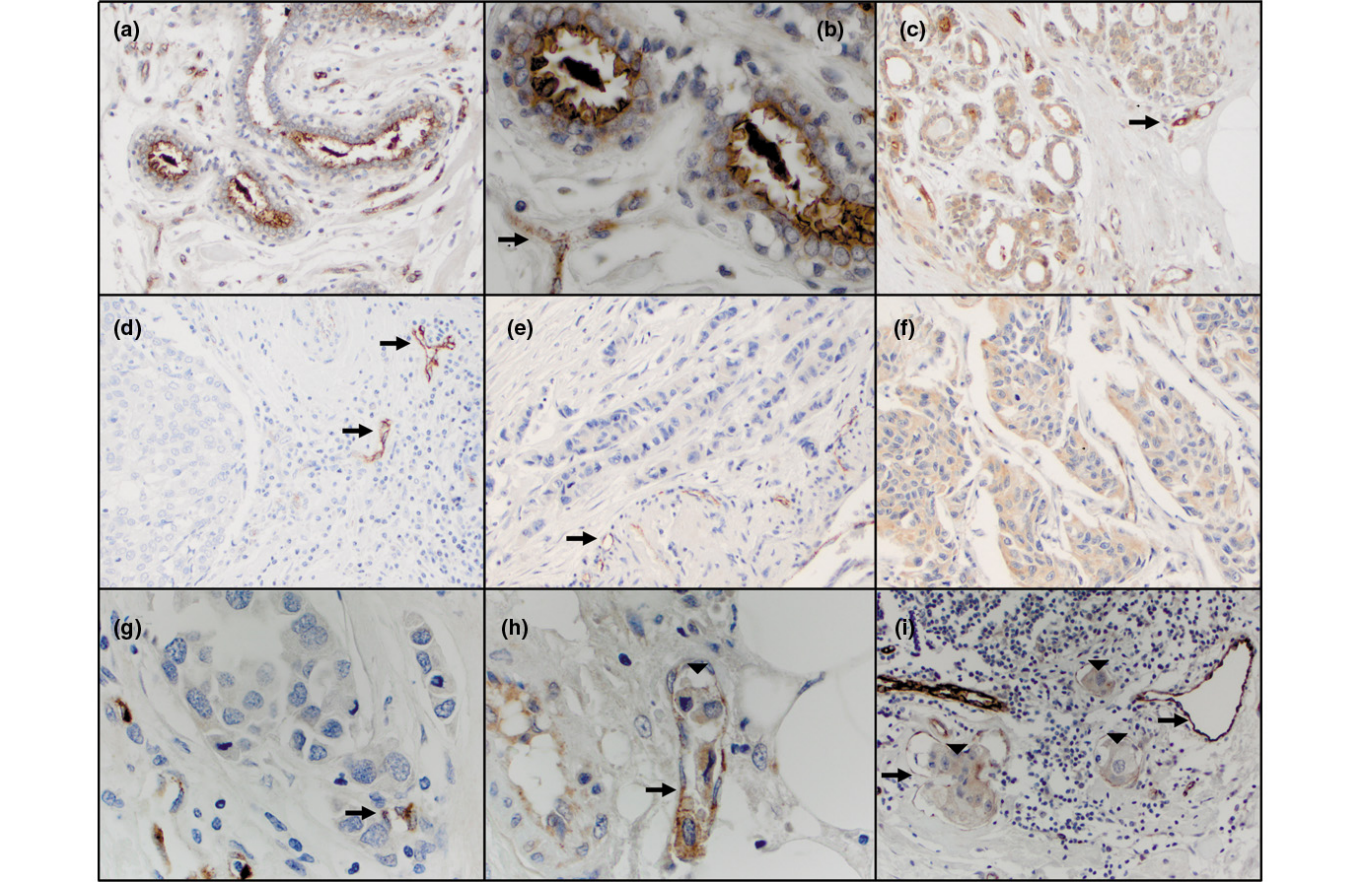
MUC4 expression in human breast tissue. Tissue microarrays were analyzed by immunohistochemistry using 1G8s. **(a) **Normal human breast of patient number 1 at 200×. **(b) **High magnification (500×) of patient number 1 normal breast, highlighting strong apical staining of MUC4 in epithelia, and endothelial staining (black arrow) as an internal control. **(c) **Normal human breast tissue of patient number 2 at 200×, exhibiting cytoplasmic staining patterns. **(d) **Matched primary invasive ductal carcinoma (200×) of patient number 2. Note the MUC4-positive blood vessels (black arrows), but the neoplastic epithelial cells are negative. **(e) **Primary invasive ductal carcinoma (200×) of patient number 3 with positive endothelial cells noted as black arrows, but no detectable neoplastic epithelial cell staining. **(f) **Matched metastatic breast carcinoma of the lymph node of patient number 3. Note increased staining intensity of MUC4. **(g) **Primary invasive ductal carcinoma (500×) of patient number 4 with many mitotic figures. **(h) **Matched metastatic tissue from patient number 4 showing a tumor embolus (500×, arrowhead). Note the MUC4-positive tumor cells within the lymphovascular space. **(i) **Metastatic breast tumor of patient number 5 (200×). Note the lymphocytes and intensely positive vessels (black arrows). Three tumor emboli are noted (arrowheads).

MUC4 expression was largely confined to the apical surface of the normal breast epithelium (Figures 6a to 6c). The observed luminal staining most likely results from shed epithelial cells or glycocalyx, common in non-lactating breast tissue [14]. Similar staining characteristics were noted in hyperplastic breast tissues (not shown). Primary breast carcinoma frequently exhibited significantly less MUC4 than normal tissue from the same patient (compare Figures 6c and 6d) using endothelial staining as an internal positive control (arrows). MUC4 localization in primary tumors was generally consistent throughout a given sample, but the pattern differed from one sample to the next. MUC4 immunoreactivity was most frequently diffusely cytoplasmic, occasionally membranous, but rarely nuclear. Expression of several mucins, including MUC4 in other tissue types, has been reported as cytoplasmic or membranous [32-34]. Often lymph node metastatic lesions exhibited higher MUC4 staining than patient-matched primary tumors (compare Figures 6e and 6f and Figures 6g and 6h). Notably, even with the relatively modest core sizes, tumor emboli were often observed within lymphovascular spaces of lymph node tissue (Figures 6h and 6i, arrowheads), where both the vasculature and the adherent epithelial emboli stained positively for MUC4.

### MUC4 promotes aggressive properties of breast tumor cells

**Figure 7:**
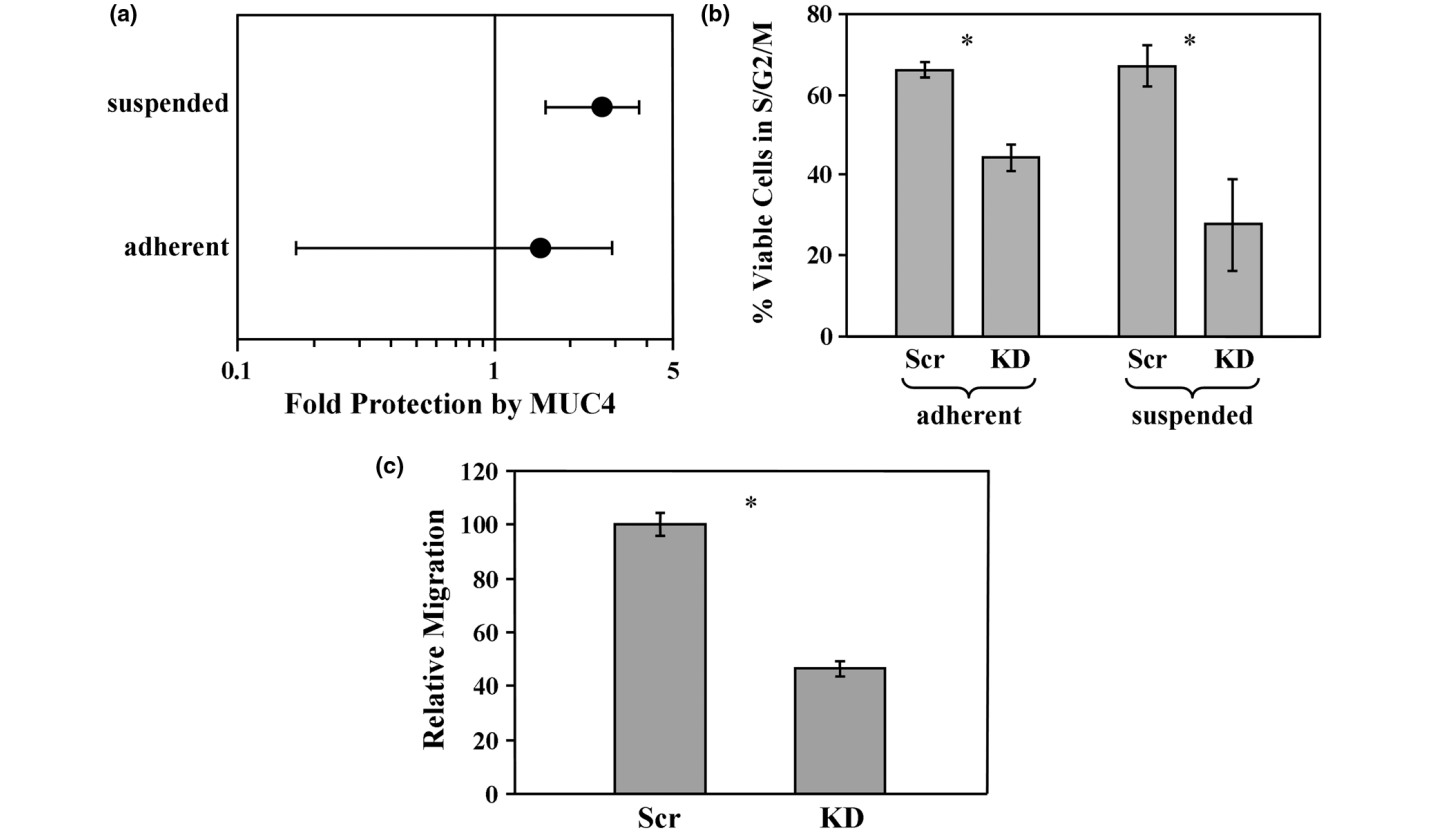
MUC4 expression promotes breast tumor cell aggressiveness. **(a) **Death of adherent or suspended JIMT-1 cells stably transduced with scrambled or MUC4-directed shRNA was measured by sub-G1 DNA content. The fold increase in the probability that the cells will undergo death in the absence of MUC4 relative to MUC4 expression was calculated and plotted for adherent and suspended conditions. 1 represents no effect, and 5 represents a 5-fold higher probability as calculated by odds ratio, with a *P *value of < 0.001. Error bars indicate the 95% confidence interval for the population, and the points indicate the center of the confidence interval. **(b) **JIMT-1 cells described in **(a) **were analyzed for DNA content by fluorescent-activated cell sorting, and the percentage of cells outside G1 phase of the cell cycle (excluding sub-G1) were plotted. **(c) **Migration of JIMT-1 cells stably transduced with scrambled and MUC4-directed shRNAs was determined by Boyden chamber assay. * *P *< 0.05.

The strong tendency for MUC4 to become re-expressed in metastatic lesions relative to matched primary tumors raises the possibility that MUC4 expression promotes cellular properties related to metastasis. To test this, we examined the impact of MUC4 knockdown on metastasis-associated properties of JIMT-1 cells. A significant barrier to metastasis is anoikis, or cell death associated with the loss of cellular adhesion to a substratum or other cells. We observed that shRNA-mediated depletion of MUC4 reproducibly elevated the probability that JIMT-1 cells will undergo death by almost three-fold when grown in suspension, but did not reproducibly affect the viability of adherent cells (Figure 7a) because essentially the entire adherent population is viable. Moreover, MUC4 knockdown limited the progression of both adherent and suspended JIMT-1 cells through the cell cycle (Figure 7b), suggesting that MUC4 may promote the proliferation of metastasizing breast tumor cells. Finally, MUC4 knockdown impaired the motility of JIMT-1 cells (Figure 7c). JIMT-1 cells appear not to be significantly invasive, so MUC4 knockdown had little impact on invasiveness (not shown). Coupled with previous observations that MUC4 is potently anti-adhesive [24] and may be capable of allowing individual cells to more easily break away from the primary tumor mass, these observations suggest that MUC4 expression also allows those cells to more easily migrate to the vasculature to initiate metastasis, survive in circulation, and proliferate when not physically attached to tissue.

## Discussion

A number of studies underscore the notion that MUC4 may be capable of contributing to the malignant properties of tumor cells. Inducible expression of rat MUC4 in human A375 melanoma cells has been demonstrated to augment primary tumor growth [25] and metastasis [26] in a nude mouse xenograft model. *In vitro *studies using the inducible rat MUC4 expression model and other cell lines indicate that ErbB2-dependent and ErbB2-independent signaling activities of MUC4 contribute to its proliferative and anti-apoptotic functions [9,11,12,35]. Moreover, the abundant O-linked glycosylation of MUC4 contributes to its anti-adhesive properties [24], masks antigens on tumor cell surfaces and inhibits cell killing by cytotoxic lymphocytes [36]. Collectively, these observations raise the possibility that dysregulation of MUC4 in patient tumors can confer properties to tumor cells that promote tumor progression.

Although ample *in vitro *and nude mouse model evidence exists that dysregulated MUC4 can potentially play a role in human tumors, evidence that it does so has been harder to obtain. The most convincing data come from pancreatic carcinomas, where normal tissue lacks MUC4 and expression increases with the progression of the disease [37]. MUC4 is commonly expressed in pancreatic tumor cell lines, and knockdown has been demonstrated to suppress pancreatic tumor cell proliferation, survival, and invasive properties [38]. However, involvement of MUC4 in the progression of other tumor types has been more difficult to assess because normal tissues express abundant MUC4 and because cell lines often rarely express the protein. In these cases MUC4 overexpression relative to normal tissue, MUC4 mislocalization in cells that have lost their polarity, or re-expression of lost MUC4 in more advanced tumor stages, can all markedly impact disease progression but can be difficult to detect and characterize.

Another significant challenge in discerning MUC4 involvement in the progression of many tumor types concerns its detection. Early studies employed *in situ *hybridization methods to detect the MUC4 message in normal and tumor tissue [39,40]. Although such studies provide information on where MUC4 protein can possibly be expressed, observations that rat MUC4 may be post-transcriptionally and post-translationally regulated by factors such as transforming growth factor-β and other basement membrane components [41] raise questions as to the extent to which this method can be used to accurately assess MUC4 protein expression by tissues. More recently immunohistochemical methods have been employed to assess MUC4 expression. The antibodies most commonly employed are 8G7, raised to a peptide within the repeating units of the O-glycosylation domain, and 1G8, originally raised to rat MUC4β but more recently demonstrated to react with human protein. In our hands, both of these antibodies obtained from commercial sources recognized bands that could not be knocked down with MUC4-specific RNA interference in immunoblotting experiments, raising the possibility that staining observed in immunohistochemical studies with these antibodies includes unrelated proteins. Moreover, as 8G7 is raised to an epitope whose post-translational modification could interfere with immunoreactivity, this antibody may preferentially recognize an underglycosylated subset of MUC4 in tissues.

In our study we develop a preparation of the 1G8 antibody that specifically recognizes MUC4β by immunoblotting, and whose immunoreactivity with cultured MUC4-positive breast cancer cells by immunohistochemical staining is markedly suppressed when MUC4 expression is knocked down. The reason underlying the difference in specificities between the commercial and hybridoma sources is unknown, but may be related to differences in antibody production. This reagent allowed us to localize MUC4 to the luminal surface of normal breast epithelium, observations that recapitulate those made with rat mammary glands [42]. Unexpectedly, we observed that MUC4 expression levels tended to be suppressed in primary tumors relative to normal tissue, whether examining patient-matched sample pairs or individual patient samples. The simplest explanation for these observations is that MUC4 expression is a marker for fully differentiated breast epithelium, and dedifferentiated breast tumor cells are impaired in their ability to support MUC4 expression. MUC4 expression is regained in many lymph node metastases relative to primary breast tumor, raising the possibility that the presence of MUC4 confers an advantage to metastasizing tumor cells. Consistent with these observations, our MUC4 knockdown experiments reveal that its expression contributes to the aggressive properties of breast tumor cells. Interestingly, a recent study found that MUC4 expression levels in primary prostate tumors is lower on average than in normal or benign hyperplastic tissue [43], although patient-matched tissues were not employed. In light of our findings it would be interesting to determine whether prostate metastases similarly regain MUC4 expression and contribute to prostate tumor cell aggressiveness. Such observations would lend support to the broader notion that MUC4 presence in metastasizing carcinomas contributes to tumor malignancy.

Collectively, the accumulated data point to a scenario where re-expression of MUC4 by a subset of primary breast tumor cells promotes their metastasis via several mechanisms. Overexpression or mislocalization of heavily glycosylated MUC4 by a subset of cells within a primary tumor population can contribute to the disruption of cell-cell and cell-matrix interactions, which in turn facilitates the migration of tumor cells away from the primary tumor and into the circulatory or lymphatic systems. Moreover, the anti-apoptotic signaling properties of MUC4 [12] can minimize the chances that primary tumor cells that have lost adhesion and are undergoing metastasis will undergo anoikis. Based on these arguments, we would predict that MUC4 overexpression might be particularly prevalent in actively metastasizing cells such as circulating tumor cells, or in metastatic cells that accumulate in abdominal or pleural effusions. Indeed, given the potent anti-adhesive properties of MUC4, these tumor cell populations could express very high levels of MUC4 protein, which may again be suppressed to some degree upon metastatic seeding of a solid target tissue.

Finally, if expression facilitates metastasis then MUC4 could ultimately serve as an independent prognostic marker of the most aggressive tumors. Patients whose primary tumors exhibit elevated MUC4 expression could be at higher risk of developing metastases than those whose MUC4 remains suppressed. However, it should be noted that MUC4 expression by a small subset of cells within the primary tumor mass may be sufficient to facilitate metastasis, and this population could easily be overlooked by immunohistochemical analysis. Moreover, as we have observed that pre-malignant atypical hyperplasias and DCIS have not yet suppressed their MUC4 expression, such studies would necessarily need to couple MUC4 expression analysis with careful pathological evaluation.

## Conclusions

The observations described here provide strong evidence that MUC4 becomes re-expressed during the transition of primary breast tumor to metastatic lesion. Moreover, MUC4 re-expression enhances malignancy by promoting the survival and proliferation of non-adherent actively metastasizing cells. These observations pave the way for the assessment of MUC4 expression in primary breast tumor samples as a marker for metastatic disease using the 1G8 antibody.

## Abbreviations

EDTA: ethylenediaminetetraacetic acid; ER: estrogen receptor; HER: human epidermal growth factor receptor; PBS: phosphate-buffered saline; PR: progesterone receptor; SDS-PAGE: sodium dodecyl sulfate polyacrylamide gel electrophoresis; TMA: tissue microarray.

## Competing interests

The authors declare that they have no competing interests.

## Authors' contributions

The study was conceived and designed by HCW, CS, and KLC III, and the majority of the experiments were carried out by HCW. Pathology analysis was carried out by HCW, ADB, and RDC. Western blotting analysis of human tumors was carried out by JKM and EQI, and RPK contributed to tumor cell growth analysis. DIY, LJTY, and KLC contributed critical reagents and advice, and LAB carried out statistical analysis of the data. All authors read and approved of the final manuscript.
